# Recognition of Emotion from Facial Expressions with Direct or Averted Eye Gaze and Varying Expression Intensities in Children with Autism Disorder and Typically Developing Children

**DOI:** 10.1155/2014/816137

**Published:** 2014-04-03

**Authors:** Dina Tell, Denise Davidson, Linda A. Camras

**Affiliations:** ^1^Department of Health Promotion, Loyola University Chicago, Marcella Niehoff School of Nursing, 2160 S. First Avenue, Maywood, IL 60153, USA; ^2^Department of Psychology, Loyola University Chicago, 1032 W. Sheridan Road, Chicago, IL 60660, USA; ^3^Department of Psychology, DePaul University, 2219 N. Kenmore Avenue, Chicago, IL 60614, USA

## Abstract

Eye gaze direction and expression intensity effects on emotion recognition in children with autism disorder and typically developing children were investigated. Children with autism disorder and typically developing children identified happy and angry expressions equally well. Children with autism disorder, however, were less accurate in identifying fear expressions across intensities and eye gaze directions. Children with autism disorder rated expressions with direct eyes, and 50% expressions, as more intense than typically developing children. A trend was also found for sad expressions, as children with autism disorder were less accurate in recognizing sadness at 100% intensity with direct eyes than typically developing children. Although the present research showed that children with autism disorder are sensitive to eye gaze direction, impairments in the recognition of fear, and possibly sadness, exist. Furthermore, children with autism disorder and typically developing children perceive the intensity of emotional expressions differently.

## 1. Introduction

A number of studies have shown deficits in face, eye gaze, and emotion processing in individuals with autism disorder (see [1–5], for reviews). It has been suggested that the deficits found for face and eye processing may underlie impairments seen in the recognition of emotion by such individuals and subsequently may contribute to their social impairments [5]. Although some studies have shown that children and adults with autism disorder show reflexive orienting to eye gaze cues (see [4], for a review), other studies have shown that these individuals look less at the eye region of the face (e.g., [6, 7]), engage in less mutual eye gaze behavior (e.g., [8]), and show more deficits in gaze following behaviors (e.g., [9]) than neurotypical individuals.

A number of critical issues remain concerning the facilitative effects of eye gaze cues on emotion recognition in children with autism disorder as well as typically developing children. According to the “shared signal hypothesis” [10, 11], when eye gaze direction is combined with the intent communicated by an emotional expression, it should enhance the perception of that emotion. That is, direct eye gaze should facilitate the processing of facially communicated approach-oriented emotions (e.g., anger and joy), whereas averted eye gaze should facilitate the processing of facially communicated avoidance-oriented emotions (e.g., fear and sadness).

In support of the shared signal hypothesis, Adams and Kleck [10] found that happy and angry expressions were more quickly identified with direct than averted eye gaze, whereas fear and sadness were more quickly identified with averted than direct eye gaze, at least in neurotypical adults. Additional research with neurotypical adults has shown that angry faces are judged “more angry” with direct eye gaze than averted eye gaze (e.g., [12, 13]). Consistent with these findings, in a developmental study, Akechi and colleagues found that typically developing children were faster at detecting a facial expression accompanying a gaze direction with a congruent emotional expression: anger with a direct gaze and fear with an averted gaze [14].

In contrast, Akechi et al. [14] found that children with autism disorder did not show this pattern of responding: eye gaze direction did not affect their reaction times in detecting angry or fearful faces, although they were just as accurate as typically developing children. According to Akechi et al. [14], these results suggest that children with autism disorder are less likely than their typically developing peers to integrate communicative signals present in the eyes with their emotional quality. Akechi et al. [14] postulated that children with autism disorder may not spontaneously integrate eye gaze direction and its communicative intent, especially in connection to emotional expressions or within social contexts.

Still, although a number of studies have examined sensitivity to eye gaze cues in adolescents and adults with autism disorder (see [3, 4], for reviews) and emotion recognition in such individuals (see [1, 2, 5], for reviews), far fewer studies have examined the effects of eye gaze direction on emotion recognition in children with autism disorder. In the Akechi et al. [14] study, only two emotional expressions, anger and fear, were presented to children. A primary goal of the present research was to explore the effects of eye gaze direction on emotion recognition in children with autism disorder and typically developing children across a greater range of emotional expressions and expression intensities. In particular, children with autism disorder and typically developing children were shown happy, angry, sad, fearful, and neutral faces with direct or averted eye gaze directions at two expression intensities (50% and 100%).

One reason for presenting this range of emotions was because past studies have found that while emotion recognition in individuals with autism disorder may be on par with neurotypical individuals for happy expressions (e.g., [15–17]), several studies have demonstrated that such individuals may show impairment in the recognition of negative emotions, including anger (e.g., [18]), sadness (e.g., [16]), and fear (e.g., [17, 19]). These studies, however, were conducted primarily with adolescents and/or adults, and not children with autism disorder. Moreover, it is possible that the developmental trajectory for emotion recognition may differ between children with autism disorder and typically developing children. While emotion recognition appears to be correlated with age in typically developing children (e.g., [20–23]); not all studies have found similar developmental improvements in children with autism disorder (e.g., [24]).

Equally important, differences between children with autism disorder and typically developing children in recognizing emotions may be less prevalent when emotional expressions are depicted at stronger or greater intensities than when less intense expressions are presented. However, the intensity of emotional expressions has only occasionally been studied as a factor affecting children's recognition of emotion. When photographs of emotional expressions of varying intensity levels were shown to a sample of 4- to 15-year-old typically developing children, these children were more accurate in matching emotions when photographs were presented at 50% expression intensity than when they were presented at 25% expression intensity, at least for fear, sadness, anger, and happy expressions [25]. In a study of both typically developing children and children with autism disorder, Mazefsky and Oswald [26] found that high-functioning children with autism disorder were less accurate than both children with Asperger's syndrome and typically developing children in perceiving emotion from low intensity voice cues, but not with high intensity cues. In a similar vein, Law Smith et al. [27] found that high functioning adolescents with autism disorder were significantly worse than typically developing adolescents at detecting surprise, anger, and disgust using dynamic facial stimuli at lower, but not higher, intensities. Finally, Wallace and his colleagues showed that adolescents with autism disorder required more intense facial expressions for accurate emotion recognition than typically developing adolescents [28].

These are important differences given that in everyday settings emotional expressions are often subtle. Only by examining a range of different emotions using both highly intense and less intense facial displays can a more accurate picture of emotion recognition abilities in children with autism disorder and typically developing children be generated.

## 2. Overview of Present Research

In light of past findings, the present research examined the effects of eye gaze direction and expression intensity on emotion recognition in children with autism disorder and typically developing children between eight and twelve years of age. Using a photograph task, children's accuracy in recognizing facial expressions of happy, angry, sad, and fear emotional expressions, along with neutral expressions, was investigated. All emotional expressions were presented with computer-generated direct or averted eye gaze at 50% or 100% expression strength. While other intensities can be presented (e.g., 25% or 75%), these intensities were selected because fewer differences were expected between more closely matched intensity rates (e.g., 0% versus 25%; 75% versus 100%) than less closely matched intensity rates (50% versus 100%). Specific predictions for each emotional expression were as follows.

1. Based on past studies that have shown that emotion recognition in individuals with autism disorder may be on par with neurotypical individuals for happy expressions (e.g., [15, 16]), it was predicted that children with autism disorder would be as accurate as typically developing children in recognizing happy expressions in all conditions (i.e., with direct and averted eye gaze and at 50% and 100% expression intensities).
2. It was predicted that children with autism disorder would recognize angry expressions at full (100%) strength as well as typically developing children. At 50% expression strength, children with autism disorder should perform more poorly than typically developing children. These findings were expected regardless of eye gaze direction and were based in part on the findings of Law Smith et al. [27], who showed that recognition of anger in adolescents with autism disorder was intact at 100% expression strength, but not at lower levels of intensity.
3. It was predicted that children with autism disorder would recognize fear expressions less well than typically developing children, regardless of eye gaze direction or expression intensity, based on past findings showing a “fear recognition impairment” in individuals with autism disorder (e.g., [17, 19]). Averted eye gaze was expected to facilitate recognition of fear in typically developing children, especially at 50% expression intensity.
4. Sad emotional expressions at 100% intensity and averted eyes were expected to be recognized equally well by children with autism disorder and typically developing children, based on the findings of Akechi et al. [14]. However, given that studies have shown that adults with autism disorder do not recognize sadness expressions as well as their neurotypical peers (e.g., [16]), children with autism disorder may not recognize expressions of sadness as well as typically developing children, especially at 50% intensity and regardless of eye gaze directions.
5. Children with autism disorder would be more likely to label neutral (no emotion) expressions with an emotion label than typically developing children. This prediction was based on the results of Kuusikko et al. [29], who showed that children with autism disorder perceived ambiguous emotional stimuli as negative emotions, whereas typically developing children did not perceive them as emotional.
6. Children with autism disorder would rate emotions with direct gaze as more intense and were expected to rate 50% emotional displays as more intense, than typically developing children. These predictions were based on the results of past studies that have shown that direct eye gaze can elicit arousing physiological effects in children with autism disorder and may even be too overstimulating for such children (e.g., [30]).

## 3. Method

### 3.1. Participants

#### 3.1.1. Children with Autism Disorder

Twenty-eight children with autism disorder were identified and recruited through specialized schools and clinical centers in Chicago, IL. One child relocated before the investigators could complete the procedures, and five children were unable to participate due to various scheduling difficulties, resulting in a final sample of 22 children with autism disorder (mean age = 10.31; age range = 8–12). Seventeen were male and 5 were female. All children with autism disorder who participated in this study were recruited from organizations that serve exclusively students with a formal diagnosis of autism disorder.

Clinical diagnosis for all children was established by medical evaluation with a developmental pediatrician and/or by a licensed clinical psychologist in accordance with the* Diagnostic and Statistical Manual of Mental Disorders*-IV [31] and the criteria for autism as outlined by IDEA (“Individuals with Disabilities Education Improvement Act of 2004”). Almost all of the children (*n* = 20) were administered the Autism Diagnostic Observation Schedule (ADOS-G; [32]) and the Autism Diagnostic Interview Revised (ADI-R; [33]) as part of their evaluation by the organization that was providing their educational/therapeutic services. The records of two children noted that they had been administered the Childhood Autism Rating Scale (CARS; [34]) and the Vineland Adaptive Behavior Scales (Vineland II; [35]), although their scores from these measures were not included in their records. Moreover, we were not able to locate these children's ADOS-G or ADI-R scores or determine if they were available. However, because these two children were attending the same program as the other children with autism disorder and because these two children did not perform differently on any measures than the other children with autism disorder, the decision was made to include their data in our sample.

Children with autism disorder were eligible to participate if they had (1) a formal and clinical diagnosis of autism disorder without comorbid conditions including ADHD, ODD, bipolar disorder, and anxiety disorder; (2) a verbal mental age (VMA) at or above 5 years; (3) English as their primary language; and (4) an IQ performance score (PIQ) greater than 75. For the latter, all children with autism disorder had been administered the WISC-III by a licensed professional within a year or less of the present study. In our sample, children with autism disorder had PIQ scores in the average range (*M* = 102.68, SD = 6.64). Children with lower PIQ scores (and VM scores below 5 years) were not recruited because of the nature of our experimental task.

According to children's records, children met the cutoff for autism disorder in the social domain, ADOS (social + communication):* M* = 11.00, SD = 4.66; ADI-R (social interaction):* M* = 20.00, SD = 6.11; in the communication and language domain (ADI-R:* M* = 12.33, SD = 4.67); and in the repetitive/stereotyped behavior domain ADOS (stereotyped behavior):* M* = 1.44, SD = 1.68; ADI-R (restricted/repetitive behaviors):* M* = 6.1, SD = 2.12. As an additional measure of severity of symptoms, parents of children with autism disorder were asked to complete the Social Responsiveness Scale (SRS; [36]). The SRS is a 65-item questionnaire designed to assess social awareness, social information processing, capacity for reciprocal social communication, social anxiety/avoidance, and autistic preoccupations and traits. It has shown good reliability (median alpha = .85) and is conceptually appropriate as it measures severity of symptoms rather than the presence or absence of them, thus capturing the spectrum-like condition of the diagnosis [36]. On average, children with autism disorder were evaluated to have moderate to severe levels of social impairment, *M*_*T*score_ = 109.25, SD = 8.49, and range = 98–124. *T*-scores above 76 are strongly associated with a clinical diagnosis of autism disorder and suggest severe interference in everyday social functioning.

#### 3.1.2. Typically Developing Children

Typically developing children (TD; mean age = 9.8 years; age range = 8–12 years) were recruited through schools in the Chicago area in order to match children with autism disorder and typically developing children on sex, chronological age, and verbal mental age. Only typically developing children who were enrolled in normal, age-appropriate classrooms were considered. Of the typically developing children recruited, 22 children with autism disorder were matched to 22 typically developing children on sex (17 males, 5 females) and chronological age (±2 months). Group differences in verbal mental age could not be completely eliminated (AD: VMA = 9.32, SD = 1.69; TD: VMA = 10.64, SD = 1.67) and were statistically controlled for the analysis of covariance. Verbal mental age was obtained from age equivalent scores from the Peabody Picture Vocabulary Test (PPVT-III; [37]).

### 3.2. Measures and Procedure

Each participating child was tested individually in a quiet area outside of the classroom at a time specified by the school. A school professional (e.g., teacher, nurse, and teacher's aide) was present at each session for all children. The experimental procedure was divided into two 20-minute sessions to accommodate children's school schedules. The first session consisted of the Emotion Recognition Task and the Emotion Expression Intensity Rating Task, and the second session consisted of the Emotion Situation Task which provided data for another study not included here and the administration of the PPVT. The procedure for each experimental task is outlined below.

#### 3.2.1. Emotion Recognition Task

**Figure 1:**
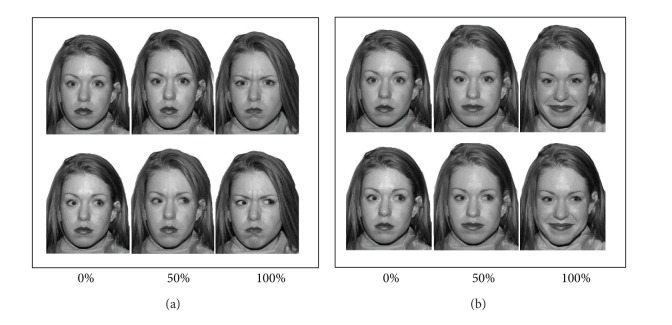
Examples of angry and happy expressions. Each emotional expression is presented as neutral, at 50% and 100% emotion intensity (from left to right) and with direct eye gaze (top) and averted eye gaze (bottom).

Facial expressions of happy, angry, sad, fear, and neutral emotions of twelve adult Caucasian actors (6 males and 6 females) were selected from the MacBrain Face Stimulus Set [38]. These high-intensity expressions were considered to be at 100% strength. To create intermediate (i.e., 50% intensity) expressions, a procedure described in Calder et al. [39] was used. This process consisted of positioning 160 points manually on the anatomical landmarks in each neutral and intense emotional photograph of the same person and combining these images using Morph X software (http://www.norrkross.com/software/morphx/MorphX.php). This resulted in the morphed intermediate expression that represented a 50% deviation in the pattern of relevant muscle movements away from the neutral expression [39]. Each emotional expression was presented at 0% (or neutral), 50%, and 100% expression strength (see Figure 1). Utilizing Adobe Photoshop software, face stimuli with direct eye gaze were computer-altered to display averted eye gaze. Averted gaze shift (right or left) was randomly assigned for each averted gaze face. Photographs were printed in black and white scale approximately 20 cm × 24 cm (see Figure 1).

#### 3.2.2. Emotion Photographs Validation Check

In order to present emotional stimuli with direct or averted eye gaze at varying expression intensities in the present study, we had to construct the stimuli ourselves, using the procedures outlined above, because none currently existed. To validate the photographic stimuli, a sample (*N* = 40) of undergraduates students at our university was asked to identify each emotional expression prior to the beginning of the study. Using frequency analysis, it was found that 98% of college students identified happy, anger, sad, and fear correctly at 100% expression strength with direct or averted eye gaze. At 50% expression strength, there was more variation: for sad and fear expressions, averted eye gaze expressions were identified correctly more often (*M* = 81%) than expressions with direct eye gaze (*M* = 70%). In contrast, happy and angry expressions were identified correctly 89%–98% of the time, respectively, regardless of eye gaze direction. Details about the validation procedure itself are available from the first author.

#### 3.2.3. Emotion Recognition Task Procedure

During the emotion recognition task, each child was presented with a set of 60 photographs of facial expressions. Forty-eight photographs included happy, angry, sad, and fearful expressions at 50% and 100% expression strengths, each with direct or averted eye gaze, and 12 photographs showed neutral faces with direct or averted eye gaze. There were a total of 12 facial expressions per emotion. To control for possible order effects, four different sets were created. Within each set, the direction of the eye gaze and expression strength were counterbalanced such that photographs with direct and averted eye gaze and 50% and 100% expression strength did not appear back to back. Potential priming effect was controlled by organizing facial expressions such that a 50% photograph never followed a 100% photograph for the same emotional expression. Each set was rotated among the participating children as enrollment progressed.

A categorization procedure in which children indicated how the person in each picture felt was used. The task started with two practice items in order to assure that children understood the nature of the task. After successful practice trials, each child was presented with the rest of the set, one photograph at a time. Photographs remained visible for as long as the child needed to identify the emotion displayed. After presenting participants with a photograph, the researcher asked* “How do you think this person feels?”* followed by presentation of a response panel of schematic faces that included verbal labels that the experimenter pointed to while saying* “Does she/he feel happy, sad, angry, scared or just ok?”* The schematic faces were black and white line drawings (4 cm × 4 cm) adapted from past research [40, 41]. The display of schematic faces was counterbalanced in order to control for biases due to a preference for a particular position. The child was allowed to either point to a schematic face or verbally state the answer. All children, however, verbally stated their answers.

#### 3.2.4. Emotion Intensity Rating Task and Procedure

A subset of 18 pictures was selected from the emotion recognition task. Two expressions (randomly selected) were used as practice items. Sixteen experimental stimuli included four emotional expressions (happy, angry, sad, and fear) at 50% and 100% of their expression strength, with direct and averted eye gaze. Using a 4-point scale, each participant was asked to judge the intensity of the emotion presented on the facial stimuli. Similar to the Hoffner and Badzinski [42] rating scale procedure, four circles increasing in size were labeled:* a little bit* (happy, angry, sad, and scared),* pretty* (happy, angry, sad, and scared),* very* (happy, angry, sad, and scared), and* very, very* (happy, angry, sad, and scared). Each picture was presented one at a time. Participants were given as much time as they needed to accomplish this task.

## 4. Results

### 4.1. Preliminary Analyses and Data Screening

Prior research suggests that the recognition of emotions might be related to overall development of verbal ability, as several studies have shown that when children with autism disorder and typically developing children were matched on verbal mental age (VMA) no differences on facial emotion recognition tasks were found [43, 44]. In the present research, VMA was obtained from age equivalent scores from the Peabody Picture Vocabulary Test (PPVT-III). An independent-samples *t*-test revealed that the difference in VMA between children with autism disorder and typically developing children was significant,* t*(42) = 2.69, *P* < .05. Therefore, in order to account for the effects of verbal ability, analysis of covariance (ANCOVA) was used with VMA as a covariate in all remaining analyses. An examination of the homogeneity of regression assumption necessary for the ANCOVA showed no violation, as evident by the nonsignificant diagnostic group (AD, TD) × VMA interactions (*F*s(1, 40) = .08–1.25, *P* ≥ .41).

### 4.2. Children's Ability to Recognize Facially Expressed Emotions: The Effects of Expression Intensity and Eye Gaze Direction

Children's mean percent accuracy scores for each emotion, across expression intensity and eye gaze directions, are shown in Table 1. To establish that children selected emotion labels for each facial expression at or above chance, goodness of fit *χ* ^2^ tests were utilized. Results indicated that children were not choosing emotions based on chance alone, *χ* ^2^ (4, *N* = 44) = 13.50–113.27, *P* < .001. A mixed 4 (emotion type: happy, angry, sad, fear) × 2 (expression intensity: 50%, 100%) × 2 (eye gaze direction: direct, averted) × 2 (diagnostic group: AD, TD) ANCOVA was performed on the accuracy scores. The Bonferroni approach was used with all post hoc pairwise comparisons in order to control for familywise error rate.

### 4.3. Recognition of Emotional Expressions: Between Group Effects at 100% and 50% Intensities

A significant diagnostic group × emotion type × expression strength × eye gaze direction four-way interaction, Λ = .80,* F*(3, 39) = 3.34, *P* < .03, and partial *η* ^2^ = .21 was found. Follow-up analyses revealed several between-group findings, as shown in Table 1. Children with autism disorder and typically developing children did not differ in their ability to correctly identify happy and angry emotional expressions at 100% and 50% strength with direct and averted eye gaze,* F*s(1, 41) = .45–2.37, *P* > .05; see Table 1. In contrast, children with autism disorder were significantly less accurate than typically developing children at identifying fear across both levels of emotion intensity (i.e., 100% and 50%), and with direct and averted eye gaze,* F*s(1, 41) = 4.68–5.81, *P* < .05; see Table 1. A trend toward significance (*F*(1, 41) = 3.65, *P* < .06) was also observed for sad emotional expressions at 100% strength with direct eye gaze, such that typically developing children were more accurate at identifying sadness than children with autism disorder.

### 4.4. Recognition of Emotional Expressions: Within-Group Effects at 100% Emotion Intensity

**Figure 2:**
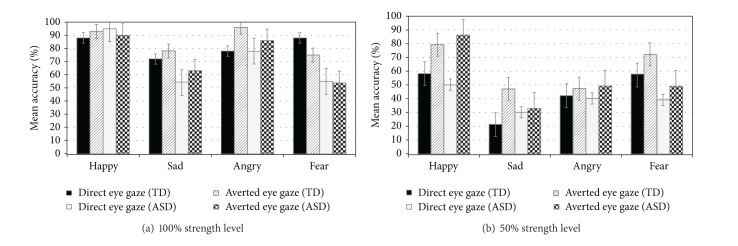
Mean percent accuracy scores for the effects of eye gaze direction on recognition of facial expressions at 100% and 50% expression strengths for children with autism disorder and typically developing children.

Pairwise post hoc comparisons were also used to examine the within-group effects of eye gaze direction on children's accuracy in recognizing each of the four emotional expressions at each intensity (Table 1 and Figure 2). In a number of instances, a different pattern of within group findings emerged for children with autism disorder and typically developing children.

Although averted eye gaze enhanced recognition of fear expressions at 100% intensity in typically developing children, it did not enhance performance in children with autism disorder,* F*(1, 39) = 5.52, *P* > .05 (see Table 1 and Figure 2). Additionally, full expressions of anger were recognized significantly better when presented with averted versus direct eye gaze in typically developing children,* F*(1, 41) = 13.88, *P* < .01, although only a trend was observed for children with autism disorder,* F*(1, 41) = 3.55, *P* < .06; see Table 1. Finally, at 100% expression strength, eye gaze direction did not influence recognition of happy expressions,* F*s(1, 41) = .09–2.49, *P* > .05, in typically developing children or children with autism disorder (see Figure 2).

### 4.5. Recognition of Emotional Expressions: Within-Group Effects at 50% Emotion Intensity

When emotional expressions were presented at 50% of their strength, typically developing children were better at recognizing expressions with averted eye gaze direction than direct eye gaze for happy,* F*(1, 41) = 10.86, *P* < .001, sad* F*(1, 41) = 13.86, *P* < .001, and fear* F*(1, 41) = 5.32, *P* < .02 expressions. Children with autism disorder benefited from averted eye gaze in the case of happy expressions,* F*(1, 41) = 32.97, *P* < .001.

### 4.6. The Effects of Emotion Type: Between and within-Group Effects

Finally, an interactive effect of diagnostic group × emotion type, Λ = .79,* F*(3, 39) = 4.89, *P* < .04, partial *η* ^2^ = .18, and two-way interaction was found for emotion type. Between-group pairwise comparisons with Bonferroni correction revealed that children with autism disorder were less accurate in their recognition of fear,* F*(1, 39) = 7.20, *P* < .02, than their typically developing peers. No group (AD, TD) differences were observed for happy, sad, or angry emotions,* F*s(1, 41) = .04–.28, *P* > .05. Typically developing children identified happy expressions with significantly greater accuracy than sadness and anger, but not fear,* F*(3, 37) = 11.65, *P* < .001. Children with autism disorder were better at recognizing happy expressions than sad, angry, and fear expressions,* F*(3, 37) = 25.04, *P* < .001; see Table 2.

### 4.7. Children's Attributions of an Emotional State to Neutral Facial Expressions

A mixed 2 (diagnostic group: AD, TD) × 2 (eye gaze direction: direct, averted) ANCOVA was performed on children's accuracy in labeling neutral facial expressions as “no emotion.” An interactive effect of diagnostic goup × eye gaze direction was found* F*(1, 41) = 20.71, *P* < .05, *η* ^2^ = .25. Children with autism disorder were less accurate at identifying neutral expressions as nonemotional when they included direct eye gaze direction (*M* = 71%, SD = 5.1), than TD children (*M* = 85%, SD = 5.2). Children with autism disorder were more likely to label a neutral facial photograph with an emotion than typically developing children (see error analysis below). No diagnostic group differences were observed for neutral expressions with averted eye gaze.

### 4.8. Error Analysis in Recognition of Facially Expressed Emotions

To examine the type of errors children made when identifying faces, emotional expressions were analyzed in terms of the frequency with which the target emotion was identified incorrectly using a 4 (error type: happy, angry, sad, fear) × 2 (expression intensity: 100%, 50%) × 2 (eye gaze direction: direct, averted) × 2 (diagnostic group: AD, TD) ANCOVAs, with diagnostic group as a between-subject factor and expression intensity, eye gaze direction, and error type as within-subject factors.

The analysis of errors revealed a significant error type × expression intensity × eye gaze direction interactions for the happy, angry, and fear emotions, Λs = .70–.79,* F*s(3, 40) = 5.10–5.45, *P* < .01, and partial *η* ^2^ = .28. Children with autism disorder and typically developing children were more likely to label happy, angry, sad, and fear expressions as neutral when faces were presented at 50% expression strength with direct eye gaze than when presented with averted eye gaze (all* P*s < .01). Children with autism disorder mislabeled sad expressions at 50% expression strength with averted eye gaze as anger more often than typically developing children. A significant main effect of error type,* F*(3, 117) = 3.98, *P* < .03, partial *η* ^2^ = .16, was found when children were viewing neutral expressions. Children across both groups confused neutral expressions with fear more so than with any other emotion.

### 4.9. Children's Ratings of Emotional Intensity: The Effects of Expression Intensity and Eye Gaze Direction

It was predicted that averted eye gaze would increase emotional intensity ratings for expressions of sadness and fear, whereas direct eye gaze would increase intensity ratings for happy and angry expressions in at least typically developing children. Interactive effects of diagnostic group × expression strength, Λ = .76,* F*(1, 41) = 14.82, *P* < .001, partial *η* ^2^ = .28, and diagnostic group × eye gaze direction, Λ = .88,* F*(1, 41) = 5.23, *P* < .02, partial *η* ^2^ = .11, were found.

While both children with autism disorder and typically developing children rated 100% expressions as more emotionally intense than 50% expressions,* F*(1, 41) = 83.45, *P* < .001,* F*(1, 41) = 12.11, *P* < .01, respectively, children with autism disorder rated facial expressions presented at 50% as more emotionally intense than typically developing children,* F*(1, 39) = 8.35, *P* < .001. Children with autism disorder also gave higher emotion intensity ratings to expressions with direct eye gaze than typically developing children,* F*(1, 41) = 10.68, *P* < .01. Within-group comparisons revealed that children with autism disorder perceived facial expressions with direct eye gaze as more emotionally intense than those with averted eye gaze,* F*(1, 41) = 22.39, *P* < .001, whereas no differences in intensity ratings between direct and averted eye gaze expressions were observed for typically developing children (see Table 3).

Intensity ratings also varied across different emotional expressions, Λ = .69,* F*(3, 39) = 5.77, *P* < .01 partial *η* ^2^ = .31, for children with autism disorder and typically developing children. Children with autism disorder rated expressions of fear as more intense than typically developing children.

### 4.10. Relation between Recognition Accuracy and SRS Scores in Children with Autism Disorder

Linear regression analyses revealed a significant negative relation between the social communication subscale and recognition of fear, *β* = −.64, *P* < .05 in children with autism disorder: children with a more severe social communication impairment were less accurate at recognizing fear than children with autism disorder who showed less impairment. A similar trend emerged for recognition of sad expressions, *β* = −.58, *P* < .07, in children with autism disorder. No other relations between the remaining SRS subscales scores and recognition accuracy (or other DVs) were found, *β*s = .04–.13,* P*s >.05. PIQ scores, VMA scores and diagnostic scores were not significantly related to children's responses (*r*s = .07–.23,* P*s > .05).

## 5. Discussion

In the present research, a number of significant findings regarding recognition of emotional expressions in children with autism disorder and typically developing children were revealed. As predicted, children with autism disorder and typically developing children did not differ in their ability to correctly identify happy and angry emotional expressions at 50% or 100% intensity with direct or averted eye gaze. However, the ability to identify fear at 50% or 100% expression intensity with direct or averted eyes was significantly less accurate in children with autism disorder than typically developing children. A trend was also found for sadness expressions: children with autism disorder were less accurate in recognizing sadness at 100% intensity and direct eye gaze than typically developing children.

The lack of difference between children with autism disorder and typically developing children in recognizing happy expressions regardless of eye gaze direction and expression intensity is consistent with the assertion that recognition of positive emotion is relatively intact in children and adults with autism disorder (e.g., [19, 45, 46]). Happy expressions are characterized by a unique mouth pattern that alone could be sufficient to discriminate them from other emotions [47]. According to Gross [46], it is possible that a visual preference for the lower part of the face accounts for the superior performance found in their recognition of happiness in individuals with autism disorder, although this cannot be determined from our data.

Also consistent with our prediction, the present study demonstrated that children with autism disorder are able to recognize expressions of anger as well as their typically developing peers. However, the expected facilitative effects of direct eye gaze on the recognition of anger were not found in either group. In fact, in typically developing children, averted eye gaze enhanced recognition of anger expressions. However, while direct eye gaze may not have improved children's recognition of anger, all children* perceived* expressions with* direct* eye gaze as more intense than expressions with averted eye gaze. This is consistent with previous research that demonstrated greater ratings of intensity for angry faces that were coupled with direct eye gaze than averted eye gaze in neurotypical adults [48].

It is not clear why averted eyes resulted in better recognition of anger expressions, as this finding is inconsistent with the shared signal hypothesis. One possibility is that children pay more attention to emotional expressions such as anger when they include averted eye gaze because they find that the same expressions with direct eyes too stimulating. This hypothesis is consistent with our findings that children rated faces with direct eye gaze, including angry expressions, as more intense than faces with averted eye gaze. It may also be that while the shared signal hypothesis holds true for adults, there may be a developmental trajectory to this effect, such that younger children do not demonstrate it. Additional research is needed to assess this assertion.

The present findings also showed that children with autism disorder were not as accurate as typically developing children in recognizing fear, regardless of expression intensity or eye gaze direction. Our results add to the growing body of literature that has reported similar “fear recognition impairments” in adults with autism disorder (e.g., [45, 49]). A number of explanations have been given to explain this impairment in recognizing fear expressions, including atypical neurological functioning in individuals with autism disorder. For example, Ashwin et al. [15, 45] showed a differential pattern of neural activity in various “social” brain areas in adults with autism disorder, compared with typical adults, during the perception of fearful facial expressions. These differences included less activation in the left amygdala and left orbitofrontal cortex brain in adults with autism disorder than in neurotypical adults.

Deficits in the recognition of fear may also be due to the fact that fear is communicated primarily by the eye region of the face and that individuals with autism disorder may pay less attention to the eyes. Although all children in this study showed sensitivity to eye gaze direction (e.g., both rated expressions with direct eyes as more intense than expressions with averted eyes for fear), averted eye gaze facilitated recognition in typically developing but not children with autism disorder. As Wallace et al. [19] note, eyes may appear differently for individual emotions, and for fear, the eyes themselves may be negatively arousing and maybe even be over arousing, for the individual with autism disorder. In expressing fear, there is a large amount of sclera visible that may lead to “fearful” eyes being perceived as threatening to the individual with autism. Thus, less attention may be paid to them [19].

Finally, our trend finding (*P* < .06) for less accurate recognition of sadness in children with autism disorder is consistent with the results of Wallace et al. [28], whose research revealed a particularly diminished sensitivity to sad expressions in adolescents with autism disorder. It is also consistent with Boraston et al.'s [16] finding of less accurate recognition of sadness in adults with autism disorder. The present results provide evidence that difficulties in recognizing fear and sad expressions may begin early in individuals with autism disorder and thus warrant further investigation.

Interestingly, negative relations between the social communication subscale of the SRS and recognition of fear and sadness were found in the present study, with children with more severe social communication impairment being less accurate at recognizing these emotions than children who showed less impairment. These results are consistent with Bal et al. [50], who showed that more severe autism disorder symptoms identified through the SRS, especially the social communication subscale, were related to less accurate recognition of emotion from dynamic (video) stimuli. Nevertheless, it is unclear whether social communicative difficulties result in less accurate recognition of negative emotions such as fear and sadness or vice versa. It has been suggested that an understanding of certain emotions such as fear requires joint communication between parents (or other adults) and children [51]. That is, the capacity to communicate, coordinate, and share attention with a social partner regarding characteristics that underlie certain emotion situations, particularly those eliciting fear or sadness, are needed for adequate perception of those emotions. For example, learning through joint attention and communication with an adult or another individual may be required in order to develop an understanding that an expression of fear is a response to a potentially threatening object in the environment.

Finally, in the present research it was found that intensity ratings for emotional expressions at 100% strength were similar for children with autism disorder and typically developing children. Moreover, both groups rated expressions presented at 100% strength as more intense than expressions presented at 50% strength. Notably, children with autism disorder rated expressions with direct eye gaze as more intense than expressions with averted eye gaze. Children with autism disorder also rated and fear emotional expressions as more intense than their typically developing peers. Children with autism disorder were also more likely than typically developing children to label neutral (no emotion) photographs with direct eyes as representing an emotion. Consistent with Kuusikko et al. [29], when children with autism disorder and typically developing children labeled neutral facial expressions with an emotion, they almost always labeled it with a negative emotion.

Although we feel these are compelling findings, limitations of this study should be noted. Firstly, we were not able to match typically developing and children with autism disorder on both chronological age and verbal mental age (VMA). However, when used as a covariate, VMA did not reveal any additional findings. Secondly, while evidence was found that children with autism disorder were sensitive to eye gaze direction, given the extensive number of manipulations in the present study, we were unable to present more than three examples per emotion of averted and direct eye gaze directions. Thirdly, while we were able to recruit all of our children with autism disorder from classrooms or programs designed to serve children with autism disorder exclusively, and ADOS-G and ADI-R scores were included in most (over 90%) children's records, we did not administer these tests ourselves. We were also not able to include a range of children on the spectrum because of the makeup of the children in the classrooms. We acknowledge that this limits the generalizability of our findings.

Finally, some have suggested that static photographical images are not ecologically valid, as emotion in everyday life is often expressed quickly and dynamically. Although static photographs may be ecologically limited, there are several reasons why the use of such stimuli in a study has value. Children's ability to recognize emotion from static images often eliminates the difficulty, and resultant floor effects, in their ability to recognize emotion from dynamic images. Additionally, emotion recognition training for children with autism disorder is often completed with static photographs of emotion. In fact, we purposely began this study before any emotion training began that year in the classroom, although we acknowledge that the children in our sample may have received emotion training in prior years. More careful inspection of the emotion training children received in a classroom or program prior to the start of research studies should be considered in future research. In spite of these limitations, an important implication of the present results is that photographs of varying expression intensities and eye gaze directions are used in emotion training, with an emphasis on presenting subtle intensities of expressions. This would provide more ecological validity with real-world emotions, to the extent that subtle displays of emotional expressions are an integral part of everyday life.

## 6. Conclusions

To conclude by highlighting our key findings, the present study showed that eye gaze direction modulates emotion perception from facial expressions in both typically developing and children with autism disorder and that children with autism disorder are sensitive to gaze direction. However, children with autism disorder showed a more significant impairment in the recognition of fear expressions and a trend for sad expressions, rated happy and fear expressions as more intense, and rated most 50% strength expressions as more intense than typically developing children. These compelling findings merit further investigation. In future research, electrophysiological and neural imaging methods with our stimuli would be valuable in shedding light on the origins of these and other differences between children with autism disorder and typically developing children in their processing of emotional expressions.

## Figures and Tables

**Table 1:** Mean percent correct values for happy, sad, angry, and fear emotional expressions as a function of expression strength and eye gaze direction.

	100% emotion strength		50% emotion strength	
	Direct	Averted	Direct	Averted
Typically developing children				
Happy	88.0 (3.6)	92.8 (4.4)	**58.0 (5.0)**	**79.5 (6.0)**
Sad	72.0 (7.2)	78.7 (7.2)	**20.9 (7.8)**	** 47.1 (5.2)**
Angry	**78.0 (4.8)**	**96.3 (4.3)**	41.6 (4.9)	47.1 (4.6)
Fear	88.1 (6.6)*	74.7 (6.0)*	57.4 (5.2)*	71.7 (8.1)*

Children with autism disorder				
Happy	94.8 (3.6)	89.6 (4.4)	**50.1 (5.1)**	**86.2 (6.0)**
Sad	54.1 (6.4)	68.5 (7.1)	30.2 (5.1)	33.6 (5.2)
Angry	77.5 (4.2)	86.2 (4.7)	40.3 (5.4)	49.5 (4.6)
Fear	54.9 (5.6)*	54.5 (6.0)*	37.8 (5.9)*	58.5 (7.0)*

Adjusted group means as percent correct values are shown. Group means are adjusted for VMA. Standard deviations are in parentheses.

*Asterisks indicate significant between-group differences (typically developing versus with autism disorder), *P* ≤ .05.

Bolded values indicate significant within-group differences (direct versus averted eye gaze), *P* ≤ .05.

**Table 2:** Mean percent correct values for happy, sad, angry, and fear emotional expressions for children with AD and typically developing children.

	Happy	Sad	Angry	Fear
Typically developing children	79.6 (4.7)	54.5 (6.9)	65.8 (4.7)	73.2 (6.4)^a^
Children with autism disorder	80.2 (4.8)	45.0 (5.9)	63.3 (4.9)	49.3 (7.1)^b ^

Adjusted group means as percent correct values are shown. Group means are adjusted for VMA. Standard deviations are in parentheses.

^a, b^Superscripts indicate significant between-group differences, *P* ≤ .05.

**Table 3:** Mean intensity ratings for facial expressions by emotion strength and eye gaze direction in children with AD and typically developing children.

Mean intensity ratings for facial expressions by emotion strength		
	100% emotion strength	50% emotion strength
Typically developing children	1.82 (.90)^a∗^	2.73 (.88)*
Children with autism disorder	2.38 (.80)^b∗^	2.73 (.89)*

Mean intensity ratings of facial expressions by eye gaze direction		
	Direct eye gaze	Averted eye gaze

Typically developing children	2.36 (.08)^a^	2.25 (.09)
Children with autism disorder	2.72 (.08)^b∗^	2.33 (.09)*

Intensity ratings ranged from 1 (least intense) to 4 (most intense). Standard deviations are in parentheses.

^a,b^Superscripts indicate significant between-group differences (typically developing versus with autism disorder), *P* ≤ .05.

*Asterisks indicate significant within-group differences, *P* ≤ .05.
